# News and Miscellany

**Published:** 1901-02

**Authors:** 


### News and Miscellany.

**A valued subscriber,** in renewing his subscription to the Journal for the seventeenth consecutive year, writes: "Death will stop my subscription to the 'Red Back,' but no other power ever will."

---

**Married**—in St. Louis, January 31, 1901, Dr. T. J. Bennett, of Austin, to Mrs. Emily J. Daniel, of St. Louis, Mo. No cards. Doctor and Mrs. Bennett will be "at home" in Austin from and after February 15th (inst.).

---

**Dr. Joseph Mullins,** of Houston, attended the Pan-American Medical Congress at Havana this month, and presented a paper on "Resection of the superior lymphatic cervical ganglion, for glaucoma," the first case in the South and the seventh in America.

---

**New Orleans Polyclinic.**—Physicians will find the Polyclinic an excellent means for posting themselves upon modern progress in all branches of medicine and surgery. The specialties are fully tought, particularly laboratory work. Fourteenth annual session opens November 12, 1900. For further information address Dr. Isadore Dyer, Secretary, New Orleans Polyclinic, New Orleans.

---

**Professor Joseph McFarland, M. D.,** the well known bacteriologist and pathologist of Philadelphia, is now *engaged prosessionally* with Parke, Davis & Co. in their bacteriological laboratory. Professor McFarland is a recognized writer, teacher, and authority on bacteriology. He has also acquired a valuable experience in practical manufacturing and executive work, whence he is now glad to escape, that he may devote himself to purely scientific research in his favorite field.

---

**Committee on National Medical Legislation.**—The American Medical Association has asked for a committee of one member from each State Medical Association to meet in Washington and appear before a congressional Committee on Public Health and urge the creation of a Public Health Department. The meeting will take place at the Arlington Hotel, February 20–21 (inst.).

Dr. B. E. Hadra, President Texas State Medical Association, has appointed Dr. J. B. Massie, of Houston, to represent the Texas State Medical Association, with Dr. Frank Paschal, of San Antonio, as alternate.

---

**Smallpox: Vaccination.**—Fluid and dry glycerinized lymph on ivory points, "both aseptic and something new," are furnished by the National Vaccine establishment at Washington, D. C. It is claimed for these points that vaccination with them does not produce sore arm. Greater convenience, time saving, increased "takes." It is aseptic. The fluid form is recommended. The manufacturers furnish both moist and dry glycerinated lymph on ivory points, packed in aseptic cases. The points are scarifiers as well as carriers of vaccine. It gives a fresh aseptic lancet for each vaccination. Process of manufacture patented, and "no other propagator can produce their glycerinized lymph without infringement of patent." We are informed that the State Health Officer uses and recommends these points.

